# Genetic Polymorphism of Cytochrome P450 4F2, Vitamin E Level and Histological Response in Adults and Children with Nonalcoholic Fatty Liver Disease Who Participated in PIVENS and TONIC Clinical Trials

**DOI:** 10.1371/journal.pone.0095366

**Published:** 2014-04-23

**Authors:** Shaminie Athinarayanan, Rongrong Wei, Min Zhang, Shaochun Bai, Maret G. Traber, Katherine Yates, Oscar W. Cummings, Jean Molleston, Wanqing Liu, Naga Chalasani

**Affiliations:** 1 Department of Medicinal Chemistry and Molecular Medicine, Purdue University, West Lafayette, Indiana, United States of America; 2 Department of Statistics, Purdue University, West Lafayette, Indiana, United States of America; 3 Department of Medical and Molecular Genetics, Indiana University School of Medicine, Indianapolis, Indiana, United States of America; 4 Linus Pauling Institute, Oregon State University, Corvallis, Oregon, United States of America; 5 Johns Hopkins Bloomberg School of Public Health, Baltimore, Massachusetts, United States of America; 6 Department of Pathology and Laboratory Medicine, Indiana University School of Medicine, Indianapolis, Indiana, United States of America; 7 Department of Pediatrics, Indiana University School of Medicine, Indianapolis, Indiana, United States of America; 8 Division of Gastroenterology and Hepatology, Indiana University School of Medicine, Indianapolis, Indiana, United States of America; 9 Indiana Fatty Liver Disease Research Group, Indiana University School of Medicine, Indianapolis, Indiana, United States of America; Institute of Medical Research A Lanari-IDIM, University of Buenos Aires-National Council of Scientific and Technological Research (CONICET), Argentina

## Abstract

Vitamin E improved liver histology in children and adults with NAFLD who participated in TONIC and PIVENS clinical trials, but with significant inter-individual variability in its efficacy. Cytochrome P450 4F2 (CYP4F2) is the major enzyme metabolizing Vit E, with two common genetic variants (V433M, rs2108622 and W12G, rs3093105) found to alter its activity. We investigated the relationship between CYP4F2 genotypes, α-tocopherol levels and histological improvement in these two trials. V433M and W12G variants were genotyped in TONIC (n = 155) and PIVENS (n = 213) DNA samples. The relationships between CYP4F2 genotypes, plasma α-tocopherol levels at baseline and weeks 48 (w48) and 96 (w96) and histological end points (overall improvement in liver histology and resolution of NASH) were investigated. As a result, the V433M genotype was significantly associated with baseline plasma α-tocopherol in the TONIC trial (p = 0.004), but not in PIVENS. Among those receiving Vit E treatment, CYP4F2 V433M genotype was associated with significantly decreased plasma α-tocopherol levels at w48 (p = 0.003 for PIVENS and p = 0.026 for TONIC) but not at w96. The w96 α-tocopherol level was significantly associated with resolution of NASH (p = 0.006) and overall histology improvement (p = 0.021)in the PIVENS, but not in the TONIC trial. There was no significant association between CYP4F2 genotypes and histological end points in either trial. Our study suggested the a moderate role of CYP4F2 polymorphisms in affecting the pharmacokinetics of Vit E as a therapeutic agent. In addition, there may be age-dependent relationship between CYP4F2 genetic variability and Vit E pharmacokinetics in NAFLD.

## Introduction

Nonalcoholic fatty liver disease (NAFLD) is one of the most common liver diseases in both children and adults, and it can lead to cirrhosis, liver failure and liver cancer [1]–[3]. Weight loss through lifestyle modification is routinely recommended for individuals with NAFLD to improve their liver disease and underlying metabolic risk factors, but weight loss is often unsuccessful. Recently, the Nonalcoholic Steatohepatitis Clinical Research Network (NASH CRN) has conducted two randomized, placebo-controlled clinical trials to improve liver histology in children and adults with NAFLD and NASH, respectively. The “Pioglitazone versus Vitamin E (Vit E) versus Placebo for the Treatment of Non-diabetic Patients with Nonalcoholic Steatohepatitis” (PIVENS) trial compared vitamin E (α-tocopherol, 800 IU per day) or pioglitazone (30 mg/day) against placebo for 96 weeks in 247 adults with NASH [4]. In this trial, Vit E improved a number of histological endpoints including a reduction in the NAFLD activity score (NAS) by ≥ 2 points as well as the resolution of NASH. The “Treatment of Nonalcoholic Fatty Liver Disease in Children” (TONIC) compared Vit E (α-tocopherol, 800 IU per day) or metformin (500 mg twice daily orally) against placebo for 96 weeks in 173 children with histologically confirmed NAFLD [5]. In this trial, Vit E significantly improved NAS and hepatocellular ballooning and resolved NASH in a significantly higher proportion of children.

Although both PIVENS and TONIC trials have shown that Vit E improves liver histology in NAFLD, there were remarkable inter-individual differences in the response to Vit E treatment in both clinical trials. The mechanism underlying this inter-individual variability in the response to Vit E remains to be addressed [6]–[10]. Recent studies have shown that cytochrome P450 4F2 (CYP4F2) is the rate-limiting enzyme responsible for the ω-oxidation of Vit E [11]–[14]. Interestingly, two common nonsynonymous single nucleotide polymorphisms (SNPs) altering amino acid coding at positions 12 and 433 of the *CYP4F2* gene have been reported to significantly influence its enzyme activity [14]–[23]. The W12G (rs3093105) substitution was reported to increase the enzyme activity by 200% [14], while the V433M (rs2108622) substitution was reported to decrease the enzyme activity by 50% [14). The V433M polymorphism is also significantly associated with warfarin dose requirement [15]–[19], and increased susceptibility to hypertension [20] and stroke [21]. These two polymorphisms were also associated with vitamin E homeostasis and its plasma concentration [22], [23]. We thus hypothesized that these two polymorphisms may affect the pharmacokinetics (PK) of Vit E and further underlie the inter-individual variability in the response to Vit E in individuals with NAFLD. In order to investigate this hypothesis, we examined the relationship between these two CYP4F2 SNPs, α-tocopherol levels before and during treatment as well as histological endpoints in PIVENS and TONIC clinical trials.

## Materials and Methods

### Ethical statement

This study represents an ancillary study of the NASH CRN and it was reviewed and approved by the steering committee of the NASH CRN. All participants signed an informed consent prior to their participation in the clinical trials and their informed consent allowed for future testing of genomic DNA for clinical investigations. DNA samples of all subjects were received for genotyping after de-identified. The current study used data previously collected from the clinical trials without further intervention with patients.

### Participants

Participant characteristics have been described in detail in previous publications [11], [12]. Subjects with available genomic DNA sample (n = 368) were included in the present study, which consisted of 213 adults from the PIVENS and 155 children from the TONIC clinical trial. Among these patients, 73 adults in the PIVENS clinical trial and 50 children in the TONIC clinical trial received Vit E for 96 weeks. The remaining participants received either placebo or pioglitazone (in case of PIVENS) or metformin (in case of TONIC).

### Genotyping

Sequences containing the CYP4F2 W12G and V433M polymorphisms were amplified by PCR. The primer sequences for PCR amplification were 5′-CAGGAAGTCCATCCATCCTGA-3′ for W12G-F;5′-GGCCTTTCTGGACTTTACCTCT-3′ for W12G-R; 5′-CTCTAGGAGCCTTGGAATGGA-3′ for V433M-F and 5′- CTCCTGACTGCTCCCTTCTCTC-3′for V433M-R. PCR reactions were set up in a 25 ul volume using the HotStar Taq Master Mix Kit (Qiagen, CA, USA) with 10 pmol of each primer and 10 ng DNA. DNA amplification was performed in the GeneAmp PCR System 2700 thermal cycler (Applied Biosystems, CA, USA) with the following program: 10 min at 95°C for 1 cycle, followed by 35 cycles of 95°C for 10 s, 58°C for 20 s and 72°C for 40 s and 1 final extension cycle of 72°C for 7 min. PCR products were then purified using the ExoSAP-IT reagent (Affymetrix, CA, USA) according to the manufacturer's instructions. The purified PCR products were directly sequenced using the BigDye Terminator v3.1 Cycle Sequencing Kit (Applied Biosystems, CA, USA) according to the manufacturer's instructions, and the sequencing results and genotypes were read and summarized using the Mutation Surveyor DNA Variant Analysis Software (Softgenetics, PA, USA).

### Data analysis

Vit E (α-tocopherol) levels before and during treatment were normalized to the corresponding measurements of total cholesterol, and the ratios were used for the subsequent analyses. For the remainder of the manuscript, α-tocopherol adjusted for cholesterol was simply referred to as α-tocopherol. Data were presented as median and range (min and max) of the ratios, while statistical analyses are based on the log transformed (+log_10_) data. The histological endpoints of interest were: 1) Overall histological improvement (yes or no), defined as at 96 weeks of treatment, whether or not there is improvement in ballooning score by ≥ 1 point, while no increase in the fibrosis score; and either a decrease of the NAS score to ≤ 3 points OR a decrease in NAS of at least 2 points with at least a 1 point decrease in either the lobular inflammation or steatosis score; 2) Resolution of NASH, defined as the absence of steatohepatitis on the week 96 liver biopsy; 3) Improvement in steatosis, lobular inflammation or hepatocyte ballooning (yes or no), defined as improvement in steatosis, lobular inflammation and hepatocyte ballooning by ≥ 1 point at 96 weeks. The analyses were performed separately for individuals participating in PIVENS and TONIC clinical trials.

Comparison of the α-tocopherol levels (log transformed ratios for baseline, at weeks 48 and 96, as well as the ratios between levels at week 48 or 96 and baseline) between PIVENS and TONIC participants (baseline for all genotyped participants, week 48, 96 measurements and the ratios for Vit E treated patients), and between each treatment arm and placebo was performed using ANOVA test. Non-parametric Kruskal-Wallis test was performed for those samples that fail to meet normal distribution assumptions. ANOVA test was also used to test the associations between α-tocopherol levels (before and during treatment) and histological endpoints. Relationships between CYP4F2 polymorphisms and α-tocopherol levels were tested using a linear regression model assuming an additive effect of the M allele at the V433M locus and a recessive model for the G allele at the W12G locus, given the very low frequency of the GG genotype (GG% = 0.013). Chi-square test or Fisher's exact test were used to examine the Hardy-Weinberg Equilibrium (HWE) and genetic associations between V433M and histological endpoints. The V433M was tested based on a 2×3 contingency table, while W12G was based on a 2×2 table after combining the GG and WG genotype groups, again due to low frequency of the GG genotype. Statistics were performed using the SPSS version 20.0 (SPSS Inc., IL, USA), SAS version 9.3 and data were plotted using the Graphpad Prism 6.0 (Graphpad Software, CA, USA). An overall α value of 0.05 was used as the cut-off for statistical significance.

## Results

### Genotype and allele frequency

The allele frequencies for V433M and W12G for all genotyped samples included in this study were 0.25 and 0.14, respectively, which are very comparable to the HapMap data (CEU population, 433M = 0.23%, 12G = 11%). There was also no significant deviation from Hardy-Weinberg Equilibrium in both loci (Fisher's exact p = 0.21 for V433M and p = 0.40 for W12G).

### Comparison of Vit E levels between PIVENS and TONIC participants

The total baseline cholesterol level of TONIC patients was significantly lower than that of the PIVENS (p<10^−4^) (Table S1). Plasma baseline α-tocopherol levels without normalizing to total cholesterol levels was also significantly lower in TONIC samples compared to PIVENS (p<10^−4^) (Table S1). After normalization, the baseline plasma level of α-tocopherol was still significantly lower in the TONIC group than in the PIVENS participants (p = 4.8×10^−5^). The normalized α-tocopherol levels were then used for subsequent analyses. This normalized α-tocopherol level during the treatment period among the individuals in the PIVENS receiving Vit E was also significantly higher than the children who were treated with Vit E in TONIC trial (p = 0.003 and 0.037 for week 48 and 96 measurements, respectively) (Table 1).

**Table 1:** Plasma α-tocopherol levels at baseline and during PIVENS and TONIC clinical trials.

PIVENS										
	Entire cohort		Vit E		Piog		PLB		Vit E vs PLB	Piog vs PLB
	Median	Min-Max	Median	Min-Max	Median	Min-Max	Median	Min-Max	p	p
Baseline α-toco	0.58*	0.23–2.00	0.61	0.28–1.16	0.57	0.26–2.00	0.56	0.23–1.01	-	-
α-toco at week 48	-	-	1.33	0.50–3.29	0.56	0.21–1.16	0.63	0.38–1.14	*<0.0001*	-
α-toco at week 96	-	-	1.22	0.49–2.48	0.55	0.21–1.17	0.59	0.27–1.22	*<0.0001*	-
Week 48/baseline	-	-	2.18	0.97–5.74	0.98	0.41–2.26	1.07	0.74–2.63	*<0.0001*	*0.0026*
Week 96/baseline	-	-	1.89	0.53–4.54	0.91	0.34–2.39	1.01	0.49–2.99	*<0.0001*	-

TONIC										
	Entire cohort		Vit E		Met		PLB		Vit E vs PLB	Met vs PLB
	Median	Min-Max	Median	Min-Max	Median	Min-Max	Median	Min-Max	p	p
Baseline α-toco	0.49	0.23–1.70	0.48	0.30–1.70	0.49	0.28–0.95	0.48	0.23–1.42	-	-
α-toco at week 48	-	-	1.08	0.43–5.06	0.53	0.26–1.87	0.51	0.31–1.30	*<0.0001*	-
α-toco at week 96	-	-	1.08	0.46–3.39	0.47	0.29–0.75	0.51	0.20–0.95	*<0.0001*	-
Week 48/baseline	-	-	2.14	0.64–5.01	1.07	0.38–3.27	1.05	0.33–2.8	*<0.0001*	-
Week 96/baseline	-	-	2.07	0.48–4.91	0.95	0.44–1.83	1.06	0.17–2.24	*<0.0001*	-

Data are shown as median and range (min-max) of plasma α-tocopherol adjusted for total cholesterol (mg/g).

*Baseline plasma α-tocopherol in PIVENS was significantly higher than the TONIC cohort (p = 4.8×10^−5^).

α-toco =  α-tocopherol; Piog = Pioglitazone; Met = Metformin; PLB = placebo.

### Comparison of Vit E levels between active treatment and placebo groups

Among individuals receiving Vit E, the plasma α-tocopherol levels increased by about 2-fold at 48 and 96 weeks relative to their baseline levels. The ratios between the measurements at week 48 or 96 and the baseline level measured before treatment were significantly higher in the patients who received Vit E compared with those who received placebo (p<0.0001 in PIVENS and <0.0001in TONIC). As expected, there was no difference in the α-tocopherol level among children treated with metformin at weeks 48 and 96, either relative to their baseline or the placebo group (Table 1). Similarly, the α-tocopherol levels did not change significantly at weeks 48 and 96 in adults in the PIVENS trial who received pioglitazone (Table 1). In order to clarify whether the difference was significantly attributed to the differences in cholesterol level, the absolute values of plasma α-tocopherol levels were also compared. A similar result was observed (Table S2).

### Relationship between α-tocopherol levels and histological response in the Vit E treatment group

Surprisingly, the α-tocopherol levels at week 96 (but not at week 48) were significantly lower among Vit E treated PIVENS adults who exhibited resolution of NASH (p = 0.006) or overall improvement in histology (p = 0.02) (Table 2). Similar results were observed for absolute values of plasma α-tocopherol levels, suggesting these associations were not affected by the cholesterol levels (Table S3). A similar pattern was not observed among Vit E treated children in the TONIC trial (Table 2). There was no significant association between α-tocopherol levels during treatment and improvement in steatosis, fibrosis, ballooning and inflammation (data not shown). There was also no significant association between the baseline α-tocopherol levels and any of the histological endpoints in either of the clinical trials (data not shown).

**Table 2:** Association between α-tocopherol level during treatment and histological endpoints among Vit E treated participants in PIVENS and TONIC.

Response	Yes		No		p value
	Median	Min-Max	Median	Min-Max	
**PIVENS**					
*NASH Resolution* *					
α-toco at week 48	1.33	0.57–2.21	1.31	0.50–3.29	-
α-toco at week 96	0.98	0.49–1.89	1.31	0.50–2.48	***0.006***
Week 48/baseline	2.29	0.97–5.74	2.05	1.42–4.9	-
Week 96/baseline	1.8	0.53–3.13	2.03	0.85–4.54	***0.006***
*Overall Improvement* *					
α-toco at week 48	1.28	0.57–2.69	1.37	0.50–3.29	-
α-toco at week 96	1.03	0.49–1.89	1.32	0.50–2.48	***0.021***
Week 48/baseline	2.22	0.97–5.74	2.09	1.42–3.83	-
Week 96/baseline	1.88	0.84–3.24	1.99	0.85–4.42	-
**TONIC**					
*NASH Resolution*					
α-toco at week 48	1.29	0.57–5.06	0.99	0.43–1.85	-
α-toco at week 96	1.09	0.50–3.39	0.82	0.47–1.63	***0.05***
Week 48/baseline	2.18	0.64–5.01	2.15	1.14–3.48	-
Week 96/baseline	2.22	0.48–3.35	1.55	1.18–4.91	-
*Overall Improvement*					
α-toco at week 48	1.11	0.43–1.91	1.07	0.48–5.06	-
α-toco at week 96	1.08	0.47–1.99	1.12	0.52–3.39	-
Week 48/baseline	2.25	0.64–4.29	2.10	1.08–5.01	-
Week 96/baseline	2.05	0.48–3.34	2.13	1.02–4.91	-

*See text for definitions.

### Relationship between CYP4F2 polymorphisms and α-tocopherol levels

**Figure 1:**
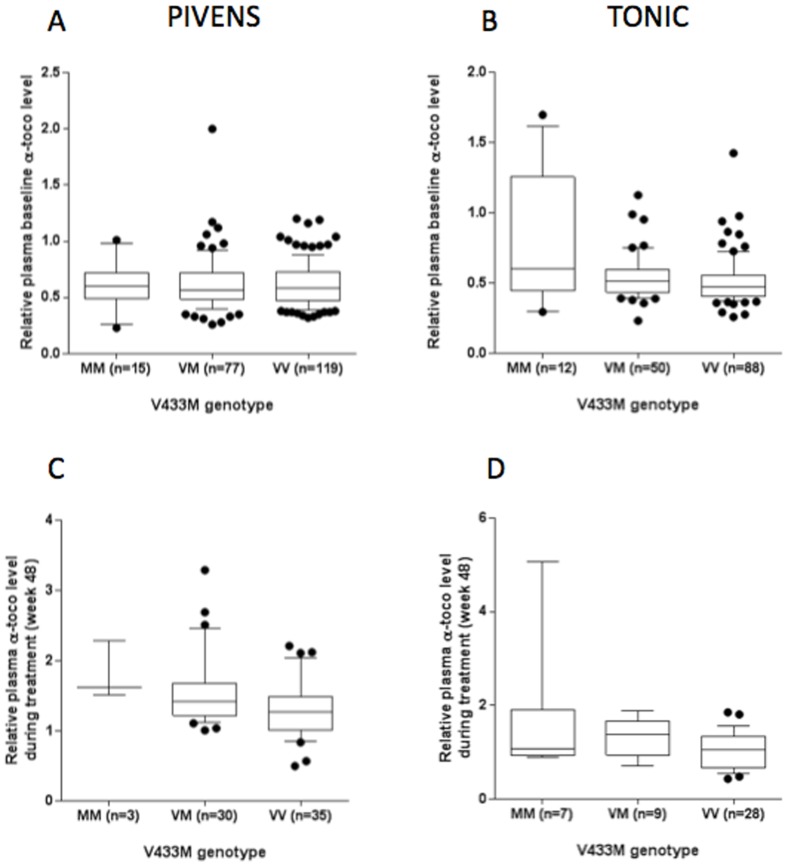
Association between CYP4F2 V433M polymorphism and α-tocopherol levels at baseline (entire cohort) and during the intervention phase (in Vit E treated participants) in PIVENS and TONIC trials. Data shown in Box (horizontal bar in the box indicates median) and whiskers plot (with 10–90 percentile). Panel A: Relationship between V433M genotype and baseline α tocopherol level in PIVENS (p = ns). Panel B: Relationship between V433M genotype and baseline α tocopherol level in TONIC (p = 0.004). Panel C: Relationship between V433M genotype and week 48 α tocopherol level in PIVENS (p = 0.004). Panel D: Relationship between V433M genotype and week 48 α tocopherol level in TONIC (p = 0.0002).

There was significant correlation between V433M genotype and baseline α-tocopherol level among children in the TONIC trial (all participants, correlation coefficient r = −0.23, p = 0.004), where the M allele was shown an additive effect on Vit E metabolism causing a higher relative α-tocopherol level (Fig. 1A and 1B). However, no such association was noted in the PIVENS trial.

Among participants receiving Vit E, the V433M polymorphism was significantly associated with higher α-tocopherol levels at week 48 in both PIVENS (r = −0.35, p = 0.004) and TONIC (r = −0.34, p = 0.026) trials (Table 3, Fig 1C and 1D). This association in PIVENS was stronger (r = −0.43, p = 0.0002), when using the ratio between the measurements at week 48 and baseline (data not shown). As shown in Table 3, there was no association between CYP4F2 genotypes and α-tocopherol levels at week 96 in either clinical trial.

**Table 3:** Association between CYP4F2 polymorphisms and plasma α-tocopherol levels.

	V433M		W12G	
	r	p	r	p
**PIVENS**				
Baseline α-toco	0.01	-	0.04	-
α-toco at week 48	−0.35	***0.003***	−0.17	-
α-toco at week 96	−0.18	-	-0.14	-
Week 48/baseline	−0.43	***0.0002***	−0.27	***0.019***
Week 96/baseline	−0.22	-	-0.20	-
**TONIC**				
Baseline α-toco	−0.23	***0.004***	−0.10	-
α-toco at week 48	−0.34	***0.026***	−0.04	-
α-toco at week 96	−0.21	-	−0.19	-
Week 48/baseline	−0.08	-	−0.05	-
Week 96/baseline	0.04	-	−0.08	-

Analyses were based on linear regression which assumed an additive effect of the 433V or 12G allele

on Vit E metabolism (e.g. assigning the M/M, M/V and V/V genotypes to 0, 1 and 2). The association

between baseline level and CYP4F2 polymorphisms was tested in all cases for each trial,

while the association during treatment was tested only in the patients treated with Vit E. The r values refer to correlation coefficients.

### Association between CYP4F2 polymorphisms and histological response

There was no significant association between either SNP and overall histological improvement or resolution in NASH in either clinical trial (Table 4). Similarly, when the analyses were limited to those who received Vit E, no significant association was observed between CYP4F2 polymorphisms and any of the histological endpoints in either clinical trial (Table 4). There were statistically significant associations noted between V433M genotypes and improvement in hepatocyte ballooning (p = 0.024) and in fibrosis (p = 0.018) in the entire genotyped cohort of the TONIC trial.

**Table 4:** Association between CYP4F2 polymorphisms and histological response.

	Response	Entire cohort							Vit E treated group						
		V433M				W12G			V433M				W12G		
		MM	VM	VV	p	GG+WG	WW	p	MM	VM	VV	p	GG+WG	WW	p
**PIVENS**															
Overall improvement	Y	3	25	44	ns	33	63	ns	2	14	15	ns	10	22	ns
	N	9	36	51		19	54		0	14	15		10	19	
Resolution of NASH	Y	5	26	41	ns	19	54	ns	1	10	15	ns	6	21	ns
	N	10	46	66		41	81		2	19	22		15	28	
Improvement in															
- Steatosis	Y	8	40	49	ns	32	66	ns	2	15	19	ns	12	25	ns
	N	7	32	58		28	69		1	14	18		9	24	
- Inflammation	Y	7	34	53	ns	28	67	ns	1	17	19	ns	11	27	ns
	N	8	38	54		32	68		2	12	18		10	22	
- Ballooning	Y	4	30	49	ns	24	60	ns	2	18	15	ns	13	23	ns
	N	11	42	58		36	75		1	11	22		8	26	
- Fibrosis	Y	7	24	39	ns	21	49	ns	1	12	15	ns	7	21	ns
	N	8	48	67		39	85		2	17	21		14	27	
**TONIC**															
Overall improvement	Y	6	16	30	ns	17	35	ns	4	4	11	ns	4	15	ns
	N	4	29	53		16	71		3	6	17		2	25	
Resolution of NASH	Y	5	13	31	ns	10	39	ns	5	6	12	ns	4	19	ns
	N	5	23	37		17	49		2	3	11		2	15	
Improvement in															
- Steatosis	Y	6	21	41	ns	19	49	ns	4	5	16	ns	3	22	ns
	N	4	25	42		14	58		3	6	12		3	19	
- Inflammation	Y	4	21	38	ns	13	49	ns	3	5	13	ns	3	18	ns
	N	6	26	45		20	58		4	6	15		3	23	
- Ballooning	Y	4	9	36	***0.024***	7	42	ns	3	4	13	ns	2	18	ns
	N	6	37	47		26	65		4	7	15		4	23	
- Fibrosis	Y	8	18	28	***0.018***	17	38	ns	5	4	7	ns	4	13	ns
	N	2	27	55		16	68		2	6	21		2	27	

## Discussion

The mechanisms underlying inter-patient variability in both pharmacokinetics (PK) and efficacy of Vit E treatment for NAFLD/NASH have previously not been investigated. Our study explored the inter-relationship between genetic factors, Vit E levels before and during treatment and histological outcomes in adults and children who participated in PIVENS and TONIC clinical trials. We found that while common *CYP4F2* functional polymorphisms especially V433M moderately affect the plasma Vit E level during treatment, they had no significant impact on the efficacy of Vit E treatment. We observed differences between adults and children patients on the associations between Vit E level during treatment and overall histological improvement and resolution of NASH. Cross-sectional comparison revealed that there were also significant differences in the baseline and on-treatment Vit E levels between adults and children in these trials. Together, these observations may suggest complex interplay between genetic variability and non-genetic factors such as age-related physiological changes and pharmacokinetics and pharmacodynamics of Vit E in NAFLD/NASH.

The Vit E treatment increased the plasma α-tocopherol levels by ∼ 2-fold at weeks 48 and 96 in both children and adults and this observation is consistent with previous reports [24]–[27]. The baseline α-tocopherol level did not predict histological outcomes, but unexpectedly we observed a significant association between histological response and *lower* plasma α-tocopherol level among the vit E treated adults in the PIVENS trial. This inverse relationship was not attributed to the normalization of α-tocopherol level to total cholesterol level during Vit E treatment, as no significant change in total cholesterol levels between baseline and week 96 measurements was observed (p = 0.43, data not shown). Since the regulation of plasma and hepatic Vit E levels in humans especially during Vit E supplementation is complex [27], it is possible that some individuals with relatively lower plasma α-tocopherol levels may have higher hepatic α-tocopherol concentration during Vit E treatment. Alternatively, plasma α-tocopherol concentration is highly regulated by α-tocopherol transfer protein (α-TTP) levels in the liver, saturation of this transfer protein might be another reason why there's relatively lower plasma α-tocopherol levels in week 96 compared to week 48 among the vit E treated adults [26]–[28].

CYP4F2 polymorphisms have been demonstrated to be functional in changing the CYP4F2 activity [14]–[23]. Notably, in a recent genome-wide association study (GWAS) involving over 4,000 individuals, V433M was identified as one of the top genetic factors affecting plasma Vit E level [22) and the association between Vit E with CYP4F2 was also anticipated in a recent systems biology review [29]. We observed that V433M (M allele) was significantly associated with higher baseline α-tocopherol level in children who participated in the TONIC trial, which is consistent with the previous reports from both *in vitro* and *in vivo* investigations [14]–[23]. This effect was further reflected by the significant association we noted between V433M and α-tocopherol levels among Vit E treated individuals in both PIVENS and TONIC trials, suggesting that CYP4F2 polymorphisms may indeed impact the pharmacokinetics of Vit E. However, the associations between V433M and various histological outcomes were not significant. This could well be due to our small size, especially in the Vit E treated arm (n = 73 and 50 for PIVENS and TONIC trial, respectively). A recent GWAS identified other genetic variants (e.g. rs964184 at the *BUD13/ZNF259/APOA5* locus) that affect the circulating level of Vit E [22]. In addition, there are also several important factors associated with Vit E absorption, transport and disposition, e.g. α-tocopherol transport protein (α-TTP) [26], [30], where certain polymorphisms in the promoter region of this gene has been depicted previously to affect the TTP gene expression [31]. Whether these genetic polymorphisms play a role in the histological response to Vit E in NAFLD remains unaddressed and should be investigated in future studies. Alternatively, because there were marginal significant associations noted between V433M genotypes and improvement in hepatocyte ballooning (p = 0.024) and in fibrosis (p = 0.018) in the entire cohort of the TONIC trial, it may be that V433M has beneficial effects on other parameters. For instance, CYP4F2 is involved in the production of 20-hydroxyeicosatethraenoic acid (20-HETE), a molecule that is proinflammatory and can induce hyperlipidemia [21], [32], [33]. CYP4F2 is also an important endobiotic metabolizing enzyme involved in the metabolism of fatty acids such as arachidonic acid, medium and very long polyunsaturated fatty acids, eicosanoids such as leukotriene B4 (LTB4), prostaglandins and lipoxins implicating its' importance in maintaining liver PUFA levels and inflammatory status [34]–[37]. Several studies have also associated CYP4F2 expression with inflammatory disorders such as celiac disease and Crohn's disease and other diseases like cardiovascular diseases, hypertension and stroke [38]–[42]. A recent study reported an association between CYP4F2 V433M polymorphisms and metabolic syndrome in cohort of Swedish male cardiovascular patients [43]. It is possible that V433M causes less inflammation and hyperlipidemia thus improves the liver histology. This further illustrates the importance of V433M polymorphism in NAFLD that warrant further investigation in a larger patient cohort.

We observed significant differences between two clinical trials in the inter-relationship between polymorphisms, Vit E level and histological response. The reasons for these differences are unclear. Besides the sample size and the dose/body weight difference, it may reflect age as an important factor affecting the absorption, distribution, metabolism and excretion (ADME) of Vit E. Due to the dynamic physiology and ontogenic variability in drug response pathway in children, ADME of many drugs in children are commonly different from that of adults. In this case, for example, with regard to the higher baseline Vit E levels in adults than in children, it is possible that adults may have slower lipoprotein turnover and thus prolonged Vit E clearance and more matured disposition than children and adolescents. Meanwhile, the disease status and pathogenesis of NAFLD/NASH in children and adults could be different as well [44], e.g. it was suggested that lower circulating Vit E may confer susceptibility to metabolic diseases in children and young adults [45]. Previous studies have also observed that young adult patients with Type I diabetes had significantly decreased ratio of α-tocopherol/lipids compared to healthy controls [46], [47]. Therefore, the developmental genetics of the Vit E pathway and the disease etiology involved in children and adults are important questions which remain to be investigated.

## Supporting Information

Table S1 **Plasma absolute α-tocopherol and AT/CHOL ratio at baseline and during PIVENS and TONIC clinical trials.**
(DOCX)

Table S2 **Comparison of absolute α-tocopherol levels between different treatments (p-values) at baseline and during PIVENS and TONIC clinical trials.**
(DOCX)

Table S3 **Association between absolute α-tocopherol level during treatment and histological endpoints among Vit E treated participants in PIVENS and TONIC.**
(DOCX)
