# Supplementary material for: Genetic Polymorphism of Cytochrome P450 4F2, Vitamin E Level and Histological Response in Adults and Children with Nonalcoholic Fatty Liver Disease Who Participated in PIVENS and TONIC Clinical Trials
Source: PLoS One. 2014 Apr 23;9(4):e95366. doi: 10.1371/journal.pone.0095366 (PMC3997354; doi:10.1371/journal.pone.0095366)
Supplement: Table S1 — Plasma absolute α-tocopherol and AT/CHOL ratio at baseline and during PIVENS and TONIC clinical trials. (DOCX) [file pone.0095366.s001.docx]

**TABLE S1 Plasma absolute α-tocopherol and AT/CHOL ratio at baseline and during PIVENS and TONIC clinical trials.**

| PIVENS | Entire Cohort* | | Vit E | | | Piog | | | PLB | | |
| --- | --- | --- | --- | --- | --- | --- | --- | --- | --- | --- | --- |
|  | **Median** | **Min-Max** | **Median** | | **Min-Max** | **Median** | | **Min-Max** | **Median** | | **Min-Max** |
| Baseline α-toco | 12.00 | 4.51-26.00 | 12.07 | | 5.41-24.37 | 12.36 | | 4.51-24.12 | 11.48 | | 4.76-26.00 |
| α-toco at week 48 | 13.41 | 3.24-64.42 | 23.78 | | 7.92-64.15 | 9.47 | | 3.24-22.18 | 10.80 | | 4.10-22.10 |
| α-toco at week 96 | 13.13 | 4.21-46.87 | 23.15 | | 8.53-46.87 | 9.95 | | 5.51-23.32 | 11.77 | | 4.21-20.14 |
| Baseline AT/CHOL | 0.58 | 0.23-2.00 | 0.61 | | 0.28-1.16 | 0.57 | | 0.26-2.00 | 0.56 | | 0.23-1.01 |
| AT/CHOL at week 48 | 0.84 | 0.21-3.29 | 1.33 | | 0.50-3.29 | 0.56 | | 0.21-1.16 | 0.63 | | 0.38-1.14 |
| AT/CHOL at week 96 | 0.79 | 0.21-2.48 | 1.22 | | 0.49-2.48 | 0.55 | | 0.21-1.17 | 0.59 | | 0.27-1.22 |
| TONIC | **Entire Cohort** | | **Vit E** | | | **Met** | | | **PLB** | | |
|  | **Median** | **Min-Max** | **Median** | **Min-Max** | | **Median** | **Min-Max** | | **Median** | **Min-Max** | |
| Baseline α-toco | 7.89 | 3.49-32.47 | 8.12 | 3.49-32.47 | | 7.90 | 4.26-19.98 | | 7.41 | 3.64-32.47 | |
| α-toco at week 48 | 9.06 | 4.66-152.53 | 16.99 | 7.32-78.92 | | 7.34 | 3.90-14.95 | | 7.84 | 4.60-19.18 | |
| α-toco at week 96 | 8.73 | 3.90-78.92 | 17.93 | 6.77-152.53 | | 8.50 | 4.66-22.56 | | 8.54 | 5.07-22.73 | |
| Baseline AT/CHOL | 0.49 | 0.23-1.70 | 0.48 | 0.30-1.70 | | 0.49 | 0.28-0.95 | | 0.48 | 0.23-1.42 | |
| AT/CHOL at week 48 | 0.71 | 0.26-5.06 | 1.08 | 0.43-5.06 | | 0.53 | 0.26-1.87 | | 0.51 | 0.31-1.30 | |
| AT/CHOL at week 96 | 0.69 | 0.20-3.39 | 1.08 | 0.46-3.39 | | 0.47 | 0.29-0.75 | | 0.51 | 0.20-0.95 | |

*Entire cohort refers to the entire genotyped samples.
