# Supplementary material for: Genetic Polymorphism of Cytochrome P450 4F2, Vitamin E Level and Histological Response in Adults and Children with Nonalcoholic Fatty Liver Disease Who Participated in PIVENS and TONIC Clinical Trials
Source: PLoS One. 2014 Apr 23;9(4):e95366. doi: 10.1371/journal.pone.0095366 (PMC3997354; doi:10.1371/journal.pone.0095366)
Supplement: Table S2 — Comparison of absolute α-tocopherol levels between different treatments (p-values) at baseline and during PIVENS and TONIC clinical trials. (DOCX) [file pone.0095366.s002.docx]

**Table S2 Comparison of absolute α-tocopherol levels between different treatments (p-values) at baseline and during PIVENS and TONIC clinical trials.**

| PIVENS | Vit E vs PLB | Vit E vs Piog | Plb vs Piog |
| --- | --- | --- | --- |
| Baseline α-tocopherol | ns* | ns | ns |
| α-toco at week 48 | <0.0001 | <0.0001 | ns |
| α-toco at week 96 | <0.0001 | <0.0001 | ns |
| TONIC | **Vit E vs PLB** | **Vit E vs Met** | **Plb vs Piog** |
| Baseline α-tocopherol | ns | ns | ns |
| α-toco at week 48 | <0.0001 | <0.0001 | ns |
| α-toco at week 96 | <0.0001 | <0.0001 | ns |

*ns refers to not significant.
