# Supplementary material for: Genetic Polymorphism of Cytochrome P450 4F2, Vitamin E Level and Histological Response in Adults and Children with Nonalcoholic Fatty Liver Disease Who Participated in PIVENS and TONIC Clinical Trials
Source: PLoS One. 2014 Apr 23;9(4):e95366. doi: 10.1371/journal.pone.0095366 (PMC3997354; doi:10.1371/journal.pone.0095366)
Supplement: Table S3 — Association between absolute α-tocopherol level during treatment and histological endpoints among Vit E treated participants in PIVENS and TONIC. (DOCX) [file pone.0095366.s003.docx]

**Table S3 Association between absolute α-tocopherol level during treatment and histological endpoints among Vit E treated participants in PIVENS and TONIC.**

| Response | Yes | |  | No | | p value |
| --- | --- | --- | --- | --- | --- | --- |
|  | **Median** | **Min-Max** |  | **Median** | **Min-Max** |  |
| PIVENS |  |  |  |  |  |  |
| *NASH Resolution* |  |  |  |  |  |  |
| α-toco at week 48 | 23.91 | 7.92-51.04 |  | 24.23 | 11.45-64.15 | - |
| α-toco at week 96 | 21.89 | 8.53-39.77 |  | 25.84 | 10.80-46.87 | ***0.016*** |
| *Overall Improvement* |  |  |  |  |  |  |
| α-toco at week 48 | 23.90 | 7.92-51.04 |  | 24.23 | 11.45-64.15 | - |
| α-toco at week 96 | 21.89 | 8.53-39.77 |  | 25.83 | 10.45-46.87 | ***0.028*** |
| TONIC |  |  |  |  |  |  |
| *NASH Resolution* |  |  |  |  |  |  |
| α-toco at week 48 | 20.45 | 9.06-152.53 |  | 15.60 | 6.77-38.57 | - |
| α-toco at week 96 | 18.59 | 8.65-78.92 |  | 15.70 | 7.32-38.82 | ***-*** |
| *Overall Improvement* |  |  |  |  |  |  |
| α-toco at week 48 | 19.58 | 6.77-40.40 |  | 16.10 | 8.31-152.53 | - |
| α-toco at week 96 | 17.35 | 7.32-38.88 |  | 17.11 | 8.22-78.92 | - |
